# Supplementary material for: Accuracy of Noninvasive Diagnostic Tests for the Detection of Significant and Advanced Fibrosis Stages in Nonalcoholic Fatty Liver Disease: A Systematic Literature Review of the US Studies
Source: Diagnostics (Basel). 2022 Oct 27;12(11):2608. doi: 10.3390/diagnostics12112608 (PMC9689671; doi:10.3390/diagnostics12112608)
Supplement: Supplementary file 1 [file diagnostics-12-02608-s001.zip › diagnostics-1938458-supplementary.pdf]

diagnostics

Supplementary

**Search strategy for diagnostic tools accuracy performed in PubMed**		
**1**	(((Nonalcoholic Steatohepatitis OR non-alcoholic steatohepatitis OR NASH) OR ("Non-alcoholic Fatty Liver Disease"[Mesh] OR nonalcoholic fatty liver disease OR non-alcoholic fatty liver disease OR NAFLD)) OR ((cirrhosis) AND (non-alcoholic))) OR ((liver fibrosis) AND (non-alcoholic))	
**2**	"diagnostic tool*" OR "diagnostic test*" OR "diagnostic procedure*" OR "non-invasive" OR "liver screen*" OR "biopsy" OR ((biochem*) AND ("test*")) OR "biomarker*" OR "biosensor*" OR "ALT" OR "alanine transaminase" OR "AST" OR "aspartate transaminase" OR "ultrasound" OR "transient elastography" OR "MRI" OR "magnetic resonance imaging" OR "APRI" OR "AST to Platelet Ratio Index" OR "FIB-4" OR "fibrosis-4 index" OR "ELF" OR "Enhanced Liver Fibrosis" OR "NFS" OR "NAFLD fibrosis score" OR "VCTE" OR "vibration-controlled transient elastography" OR "MRE" OR "Magnetic Resonance Elastography" OR "Fibro Scan" OR "FibroScan" OR "Fibro Sure" OR "FibroSure" OR "Fibromax" OR "liver enzyme*" OR "GGT" OR "gamma glutamyl transferase" OR "NIS-4" OR "NIS4" OR "Liver MultiScan" OR "MRI-PDFF" OR "MRI proton density fat fraction" OR "Proton density fat fraction" OR "Velacur" OR "liver examination" OR "Diagnostic Tests, Routine"[Mesh] OR "Elasticity Imaging Techniques"[Mesh] OR "Magnetic Resonance Imaging"[Mesh] OR "Ultrasonography"[Mesh] OR "Biopsy"[Mesh]	
**3**	"sensitivity and specificity"[MeSH Terms] OR "sensitivity" OR "specificity" OR diagnostic test accuracy OR "test accuracy" OR "ROC curve" OR "positive predictive value" OR "negative predictive value" OR "Receiver operating characteristic"	
	**Total with limits applied (English language and humans, publication year 2016-2022)**	1,286
**Search strategy for diagnostic tools accuracy performed in Web of Science**		
**1**	(((("Nonalcoholic Steatohepatitis" OR "non-alcoholic steatohepatitis" OR NASH) OR ("nonalcoholic fatty liver disease" OR "non-alcoholic fatty liver disease" OR NAFLD)) OR ((cirrhosis) AND (non-alcoholic))) OR (("liver fibrosis") AND (non-alcoholic)))	
**2**	("diagnostic tool*" OR "diagnostic test*" OR "diagnostic procedure*" OR “non-invasive” OR "liver screen*" OR biopsy OR ((biochem*) AND (test*)) OR biomarker* OR biosensor* OR “ALT” OR "alanine transaminase" OR “AST” OR "aspartate transaminase" OR ultrasound OR "transient elastography" OR “MRI” OR "magnetic resonance imaging" OR “APRI” OR "AST to Platelet Ratio Index" OR “FIB-4” OR "fibrosis-4 index" OR “ELF” OR "Enhanced Liver Fibrosis" OR “NFS” OR "NAFLD fibrosis score" OR “VCTE” OR "vibration-controlled transient elastography" OR MRE OR "Magnetic Resonance Elastography" OR "Fibro Scan" OR FibroScan OR "Fibro Sure" OR FibroSure OR Fibromax OR "liver enzyme*" OR “GGT” OR "gamma glutamyl transferase" OR NIS-4 OR NIS4 OR "Liver MultiScan" OR MRI-PDFF OR "MRI proton density fat fraction" OR "Proton density fat fraction" OR Velacur OR "liver examination" OR Ultrasonography)	
**3**	("sensitivity" OR "specificity" OR "diagnostic test accuracy" OR "test accuracy" OR "ROC curve" OR "positive predictive value" OR "negative predictive value" OR "Receiver operating characteristic")	
	**Total with limits applied (English language and humans, publication year 2016-2022)**	1,397
